# When Employee Mental Health Deteriorates: Examining the Relationship Between Health-Oriented Leadership, Disclosure, and Sickness Absence

**DOI:** 10.3390/healthcare13212759

**Published:** 2025-10-30

**Authors:** Sarah Pischel, Jörg Felfe, Lene S. Fröhlich

**Affiliations:** Department of Work, Organizational and Business Psychology, Helmut Schmidt University Hamburg, 22043 Hamburg, Germany; felfe@hsu-hh.de (J.F.); froehlich.lene@hsu-hh.de (L.S.F.)

**Keywords:** health-oriented leadership, mental health, warning signals, disclosure, sickness absence

## Abstract

**Background/Objectives**: Given the high prevalence of mental health problems in the workplace, fostering disclosure and reducing sickness absence are critical for ensuring timely support and sustaining employees’ work ability. Drawing on the health-oriented riented leadership (HoL) model, this paper examines the associations between staff care, disclosure, and sickness absence, and addresses the underexplored question of whether staff care continues to show beneficial relationships when employees experience acute health deterioration. To account for differing perspectives, we included samples with employees and with leaders. **Methods**: We conducted three distinct cross-sectional studies with (1) predominantly healthy employees (N_1_ = 148), (2) employees with severe mental health issues or a diagnosis (N_2_ = 338), and (3) leaders (N_3_ = 91). **Results**: Staff care is positively related to disclosure across all studies. In study 1, this relationship was unexpectedly stronger for low than for high health deterioration, though still significant for high deterioration. In studies 2 and 3, the interaction was non-significant. However, a perceptual gap emerged: simple slopes showed that leaders with low staff care still expected disclosure from employees with high health deterioration (study 3), whereas employees reported higher concealment intentions (study 1). Staff care was negatively related to sickness absence only in study 2, with this relationship strengthened under high health deterioration. **Conclusions**: Staff care seems particularly relevant for supporting disclosure during early health declines and for mitigating sickness absence during acute deterioration among those already affected. Divergent leader–employee perceptions may hinder timely support. We provide practical recommendations for organizations.

## 1. Introduction

Mental health issues are associated with serious negative consequences such as increased costs, sickness absence, and reduced productivity across Europe [1,2,3]. The workplace plays a key role in both the development and management of mental health issues and illnesses [4,5], and leaders, in particular, have a significant impact on employees’ mental health [6,7]. In recent years, health-oriented leadership (HoL) has gained significant attention for its focus on health-specific attitudes and behaviors [8]. The HoL concept emphasizes that leaders actively foster employees’ well-being (staff care) by prioritizing health, being aware of warning signals, and providing explicit health-related support (e.g., offering assistance, creating healthy work conditions [9]).

A growing body of research demonstrates that staff care is related to better employees’ health-related outcomes (e.g., fewer psychosomatic complaints, lower depression, burnout, and anxiety) and more positive work-related attitudes (e.g., higher engagement; see [8] for a review). However, staff care becomes particularly demanding when employees’ health is deteriorating or when they already experience mental illness. In these situations, employee disclosure is essential for leaders to provide timely and tailored support and to prevent further declines in health and increased sickness absence. Yet, many employees conceal their struggles, which can heighten stress and reinforce a downward spiral [10,11,12,13]. Concealment may also cause leaders to misinterpret employees’ behavior and respond with pressure rather than support [14].

While previous studies show that staff care strengthens disclosure intentions and is negatively related to sickness absence [15,16], previous research has neglected employees with deteriorating mental health or mental illness, which presents additional challenges for both leaders and employees. Leaders often face significant uncertainty regarding how to respond appropriately, balancing support and privacy while avoiding “overcaring” [14,17]. Simultaneously, affected employees may struggle with shame, self-stigmatization, and withdrawal tendencies [18,19], which can hinder open communication. It remains unclear if staff care continues to relate to higher disclosure and lower sickness absences when employees’ health is at high risk or when employees already experience a mental illness. Although one might assume that employees facing severe mental health challenges are less likely to disclose their condition regardless of their leaders’ health orientation because they fear stigma and negative consequences [11,15], we propose the opposite pattern. When employees encounter deteriorating mental health, their willingness to disclose may depend on the degree of staff care they perceive from their leaders. Leaders who display high staff care may convey understanding and support rather than potential risks, which can encourage openness. Such disclosure, in turn, may alleviate unpleasant feelings and facilitate tailored support and work adjustments [11,20,21], potentially contributing to reduced sickness absence. In sum, the link between staff care, disclosure, and sickness absence may be particularly pronounced among employees experiencing deteriorating mental health. This reasoning aligns with previous findings suggesting that staff care becomes especially relevant in demanding situations [22].

When examining whether employees’ health deterioration strengthens the positive association between staff care and employees’ willingness to disclose, it is crucial to consider potential perceptual differences between leaders and employees. For instance, leaders with low health orientation may assume that employees will disclose regardless of leadership behavior, whereas employees may only feel willing to disclose when they perceive their leaders as health-oriented. Understanding these contrasting viewpoints is essential to determining whether leaders overestimate the likelihood of disclosure within their current leadership approach.

**Figure 1:**
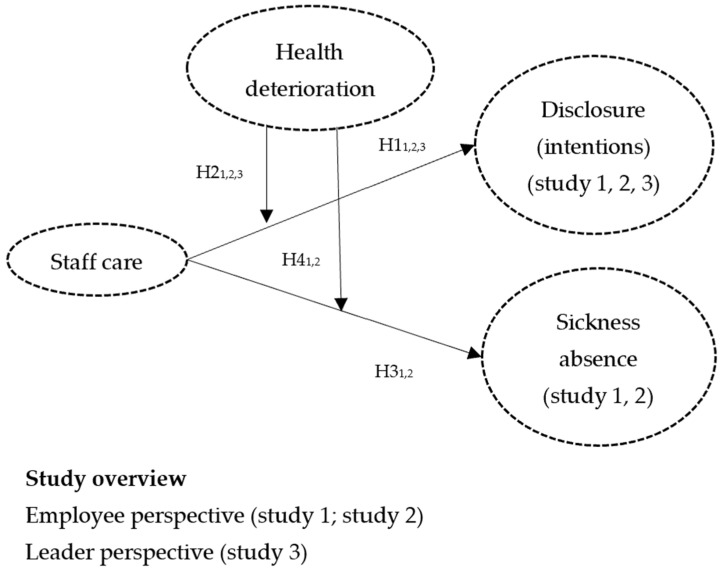
Research model.

This research addresses four main objectives (see Figure 1 for the research model). First, it examines the associations between staff care, disclosure, and sickness absence, thereby contributing to the growing evidence on the benefits of health-oriented leadership [8,9]. Second, it explores whether these relationships hold when employees experience deteriorating mental health. To the best of our knowledge, this is the first study to examine both disclosure intentions and actual disclosure behavior among employees diagnosed with a mental illness. Third, it incorporates both employee and leader perspectives to capture potential perceptual differences. Finally, the study seeks to provide practical recommendations for organizations and leaders aiming to foster openness in their workforce while minimizing disruptions caused by health-related absences.

By addressing these objectives, the study offers both theoretical and practical contributions. Theoretically, it advances our understanding of the situational contingencies for health-oriented leadership by investigating whether negative dynamics in employees’ health moderate the effects of staff care on disclosure and sickness absence. Exploring such moderators helps refine theory in health-promoting leadership by embedding leadership within a broader conceptual framework [23]. In addition, by investigating specific leadership behaviors and their interplay with employee disclosure, our work extends previous disclosure models [11,20,24]. Practically, the findings may inform leadership training programs and organizational health policies, enabling leaders to support employees’ health while minimizing productivity losses associated with sickness absence.

## 2. Theory

### 2.1. Health-Oriented Leadership and Employee Health

Leadership is increasingly recognized as a key factor in protecting and promoting employee health [6,7,25]. Research has shown that positive leadership styles (e.g., transformational leadership, relations-oriented leadership, task-oriented leadership, leader–member-exchange) are associated with better employee health, whereas destructive leadership styles (e.g., abusive supervision) are related to poorer employee health [6,26]. However, traditional leadership approaches often lack specificity regarding health-related attitudes and behaviors, as their primary focus is on performance and motivation [9].

The HoL concept [9] was developed to address this gap. It defines staff care as leaders’ genuine concern for employee health, encompassing three dimensions: value (prioritizing health), awareness (recognizing warning signals), and behavior (taking health-related action, e.g., improving work organization, promoting participation in occupational health promotion, or promoting recovery). Research has shown that staff care predicts employee health beyond transformational leadership, underscoring its unique contribution [9,15,27]. Staff care represents an external resource following Conservation of Resources (COR) theory [28], assumed to positively influence employee health. The model also highlights leaders’ self-care (i.e., leaders’ and employees’ health-oriented self-leadership) as equally important. Both staff and self-care have been shown to improve general well-being and reduce depression, anxiety, and burnout [9,29,30,31,32,33].

The present research comprises three studies (see Figure 1). Study 1 includes predominantly healthy participants and examines how staff care relates to disclosure intentions and sickness absence, particularly during early health deterioration (i.e., early warning signs). Study 2 involves participants already diagnosed with mental illness or reporting severe mental health issues and investigates the relationship between staff care, actual disclosure, and sickness absence, as well as whether these relationships vary with acute health deterioration (i.e., crises). Study 3 includes leaders and captures their perspectives on staff care, employees’ health changes, and expectations regarding disclosure, aiming to identify potential discrepancies compared to the employee perspective (study 1). In the following theoretical section, we present the hypotheses thematically according to their underlying constructs (H1–H4), while specifying the respective study in which each hypothesis was tested (e.g., H1_1_, H1_2_, and H1_3_).

### 2.2. Staff Care and Employees’ Disclosure of Mental Health Issues

Given the high prevalence of mental stress and illnesses in the workplace, disclosure has become an important research focus [20,24,34]. Disclosure refers to the voluntary revelation of mental problems or illnesses by employees to their leader [35]. It can yield substantial individual and organizational benefits, as it enables timely assistance and accommodations from leaders [36]. When employees choose not to disclose mental health issues to their leader, the leader may misinterpret observable changes (e.g., a decline in work performance as a lack of motivation), potentially leading to increased pressure and further exacerbating the employee’s distress [10,11,14].

An initial study linking staff care and disclosure research found that staff care has a positive effect on disclosure intentions in an unselected sample [15]. Replicating this finding, we expect staff care to be positively associated with disclosure intentions among predominantly healthy employees [15].

**H1_1_:**  *Staff care is positively related to disclosure intentions (study 1).*

While disclosure intentions often predict future behavior, they do not always translate into actual disclosure (intention–behavior gap; [37]). To assess whether staff care relates to actual disclosure, it is essential to move beyond predominantly healthy populations and examine employees currently facing serious mental health problems or a clinical diagnosis. Building on disclosure models, which were originally developed to explain actual disclosure among psychologically affected individuals [20,21], we assume that perceiving a caring and health-oriented leader is associated with anticipating more benefits than costs, which in turn may be linked to greater actual disclosure.

**H1_2_:**  *Staff care is positively related to actual disclosure (study 2).*

Previous research has shown that employees and leaders often hold divergent perceptions of the same workplace phenomena, including leadership behavior and its impact (e.g., [32,38]. For instance, leaders who demonstrate relatively low levels of staff care may still assume that employees will proactively disclose mental health issues to them, even without visible health-oriented behaviors. In contrast, struggling employees may intend to disclose only when they perceive genuine support and concern from their leader. Since concealment intentions, once formed, can persist over time, influence colleagues’ disclosure decisions, and contribute to further health declines [15], identifying such perceptual discrepancies during this early phase is crucial. Such insights can help raise leaders’ awareness of potential overestimations and encourage more health-oriented leadership behavior.

Drawing on the false consensus effect [39], we suggest that leaders may overestimate employees’ willingness to disclose, assuming that others would behave similarly to themselves. Thus, we expect the positive relationship between staff care and disclosure intentions to be weaker from the leader’s perspective than from the employee’s perspective.

**H1_3_:**  *Staff care is positively related to disclosure intentions (study 3).*

### 2.3. The Effectiveness of Staff Care When Facing Employees with Health Deterioration

Besides the positive association between staff care and disclosure intentions, it is essential to investigate contextual factors and boundary conditions that may shape this relationship [23]. Until now, it remains unclear whether staff care continues to relate to higher disclosure willingness when negative dynamics unfold among previously healthy employees. The initial manifestation of such negative health changes (e.g., declines in performance, mood changes, or withdrawal behaviors) is referred to as warning signals, which may develop into more severe mental illnesses over time [40].

In this situation, leaders may struggle to assess the severity of these changes and balance support with professional boundaries [14,17]. Simultaneously, employees with deteriorating mental health may experience shame, withdrawal tendencies, or denial [18,19]. This mutual uncertainty can reduce open communication and may limit the effectiveness of staff care. However, building on COR theory [28], we assume that employees facing mental health challenges have a greater need for supportive leadership and rely more strongly on external resources to maintain stability and well-being. When they perceive high staff care (e.g., leaders showing knowledge about mental health, genuine concern, and suggesting specific health-promoting measures), they may anticipate understanding and support rather than potential risks such as stigma or negative career consequences. Prior research indicates that staff care is particularly relevant in demanding situations [22]. In sum, the positive relationship between staff care and disclosure intentions may be stronger among employees with higher levels of health deterioration.

**H2_1_:**  *The positive relationship between staff care and disclosure intentions is moderated by employees’ health deterioration. The relationship is stronger at higher levels of health deterioration (i.e., initial warning signals; study 1).*

In psychologically affected populations, health deterioration may manifest as an acute episode, relapse, or escalating crisis, rather than a chronically stable condition [1,41]. While chronically stable symptoms often indicate a sustained high burden of illness, they are typically marked by relative functional stability over time [ibid.]. In contrast, acute worsening is characterized by rapid symptom escalation, heightened emotional distress, and an increased need for external support. Building on the reasoning above and COR theory [28], we expect the positive relationship between staff care and disclosure to be stronger among employees with higher levels of acute worsening.

**H2_2_:**  *The positive relationship between staff care and actual disclosure is moderated by employees’ health deterioration. The relationship is stronger at higher levels of health deterioration (i.e., acute worsening; study 2).*

While employees’ health deterioration may strengthen the link between staff care and disclosure intentions from the employee perspective, this pattern could differ from the leader’s perspective. As noted above in relation to the false consensus effect [39], leaders may assume that employees’ willingness to disclose exists regardless of their own health-specific behavior, and such beliefs may become even stronger when they observe signs of health deterioration. Consequently, we expect the positive relationship between staff care and disclosure intentions to be weaker at higher levels of health deterioration.

**H2_3_:**  *The positive relationship between staff care and disclosure intentions is moderated by employees’ health deterioration. The relationship is weaker at higher levels of health deterioration (i.e., initial warning signals; study 3).*

### 2.4. Staff Care and Sickness Absence

In addition to disclosure intention, sickness absence, i.e., the absence from work due to illness, is an important objective indicator of employee health. Sickness absence is associated with productivity losses, replacement costs, and increased strain on remaining team members [42,43,44]. Reducing sickness absence is therefore a key goal of occupational health management and sustainable organizational performance. Recent data from a large German health insurance provider show that mental illnesses are the third most common cause of sickness absence, with an average duration of 28.1 days per case, and that sickness days due to mental illness have increased by 56.5% since 2013 [45]. Even without a diagnosed mental illness, about one in four employees report having been absent due to stress or work pressure [46].

Empirical research on health-oriented leadership and sickness absence remains scarce. Initial evidence from Pundt and Felfe [16] suggests a negative association between staff care and sickness absence in a predominantly healthy sample. Leaders who prevent excessive overtime, encourage recovery, and support participation in health promotion initiatives may help maintain employee well-being. Replicating this finding, we expect staff care to be negatively associated with sickness absence.

**H3_1_:**  *Staff care is negatively related to sickness absence (study 1).*

While this association has been shown in healthy populations [16], less is known about whether it applies to employees with serious mental health problems or a diagnosis. Because such employees generally show higher absence rates [45], identifying potential links to leadership is crucial. Drawing on prior research linking positive leadership to lower absence in vulnerable groups [47,48], we expect that staff care to be negatively associated with sickness absence among psychologically affected employees.

**H3_2_:**  *Staff care is negatively related to sickness absence (study 2).*

### 2.5. The Effectiveness of Staff Care When Facing Employees with Health Deterioration

When employees exhibit warning signals, they are likely to face a higher risk of resource depletion, increasing their likelihood of sickness absence. In this context, staff care behaviors such as expressing genuine concern for the employees’ health and providing adapted support (e.g., optimizing work processes, allowing more flexible working hours, or encouraging participation in occupational health programs) may signal a resource-rich and supportive work environment [28]. We therefore assume the negative relationship between staff care and sickness absence to be stronger at higher levels of health deterioration.

**H4_1_:**  *The negative relationship between staff care and sickness absence is moderated by employees’ health deterioration. The relationship is stronger at higher levels of health deterioration (i.e., initial warning signals; study 1).*

Building on our previous reasoning and COR theory [28], employees undergoing an acute episode or crisis face intensified resource loss and may rely heavily on external support. Thus, we also expect the negative relationship between staff care and sickness absence to be stronger among affected employees with higher levels of acute worsening.

**H4_2_:**  *The negative relationship between staff care and sickness absence is moderated by employees’ health deterioration. The relationship is stronger at higher levels of health deterioration (i.e., acute worsening; study 2).*

## 3. Methods

### 3.1. Sample and Procedure

All three distinct cross-sectional studies were conducted as online surveys, with participants recruited by a market research institute as part of a larger project. Sampling followed quota criteria to approximate the German working population in terms of age and gender. All participants were employed full-time. Invitations were randomly distributed until the target sample size was reached. The number of refusals was not recorded. Participation was voluntary and anonymous.

In study 1 (*N*_1_ = 148), participants without a mental illness or severe mental health issue were eligible. The mean age was 40.81 years (*SD* = 11.46); 53.4% were female. Regarding education, 43% held a university degree and 27% a degree from vocational training or a university of applied sciences. Most participants worked in the private sector (76%), and nearly half (47%) were employed in large organizations (>500 employees). Main sectors included public administration (14.2%), IT and telecommunications (11.5%), metal and electrical industry (10.8%), and finance (10.8%).

In Study 2 (*N*_2_ = 338), participants either reported a diagnosed mental illness (49%) or a severe mental health issue without diagnosis (51%) (e.g., “I have no diagnosis of a mental illness but I do have serious mental health problems and have already started to worry about myself.”). Among those with a diagnosis, 44% reported depression, 27% comorbid mental illnesses (e.g., depression and anxiety disorders), 18% anxiety disorders, and 17% other diagnoses. The mean age was 38.25 years (*SD* = 10.58); 56.8% were female. Regarding education, 41% held a university degree and 23% a degree from vocational training or a university of applied sciences. Most participants worked in the private sector (69%), and nearly half (42%) were employed in large organizations (>500 employees). Main sectors included public administration (12.1%), IT and telecommunications (10.7%), metal and electrical industry (8.3%), logistics/transport (7.7%), and finance (7.1%).

In Study 3 (*N*_3_ = 91), leaders were asked to assess a team member they knew well and not experiencing a (known) mental illness or severe mental health issue. The mean age of leaders was 42.38 years (*SD* = 11.17, range 21–69); 60.4% were male. Regarding education, 62% held a university degree, 14% a PhD, and 11% a degree from vocational training or a university of applied sciences. Most leaders worked in the private sector (90%), and 40.7% were employed in small organizations (<50 employees). Main sectors included IT and telecommunications (24.2%), trade (9.9%), consulting (9.9%), health (8.8%), and finance (8.8%).

### 3.2. Measures

Staff care was assessed using the employee- and leader-rating staff care questionnaire of the HoL instrument [16]. For parsimony, we used a reduced scale comprising six items for behavior (e.g., “My supervisor tries to reduce my demands by optimizing my work-life balance (e.g., take regular breaks, avoid overtime, avoid the expiration of vacation days)” or “I try to reduce my followers’ demands by optimizing their work-life balance (e.g., take regular breaks, avoid overtime, avoid the expiration of vacation days)”), three items for awareness (e.g., “My supervisor consciously pays attention to my health warning signals” or “I consciously pay attention to health warning signals of my followers”), and three items for value (e.g., “My health is important to my supervisor.” vs. “My followers’ health is important to me”). All items were answered on a five-point Likert scale from 1 (not at all true) to 5 (completely true). A total score was computed across subscales. Cronbach’s α was 0.95 in study 1, 0.95 in study 2, and 0.93 in study 3.

Employees‘ current health deterioration was measured using the original Early Warning Indicator for Mental Health (EWI; [40]) instrument. The self-rating version contains 52 items, and the external-rating version includes 45 items, each covering seven subscales: depressiveness (self-rating, e.g., “Compared to before, I feel sadder (e.g., I cry more easily, laugh less)”; external-rating, e.g., “I have noticed that the employee seems to be sad more often compared to before (e.g., cries more often, laughs less)”), performance decline, social withdrawal, social incompatibility, lack of self-care, self-endangering behavior, and overcommitment. All items were answered on a five-point Likert scale from 1 (not at all) to 5 (very strong). A total score was computed across subscales. Cronbach’s α was 0.98 in study 1, 0.95 in study 2, and 0.99 in study 3.

Disclosure intentions in study 1 were measured with five items (e.g., “I would tell my leader about my mental health problem.”) adapted from the study by Pischel and Felfe [15]. Participants were asked to imagine experiencing a mental health issue and to indicate their intention on a five-point Likert scale from 1 (not at all true) to 5 (completely true). Cronbach’s α was 0.91.

Actual disclosure to leaders in study 2 was measured with one item (“I told my leader about my mental illness/severe mental health issue”) on a five-point Likert scale from 1 (told nothing) to 5 (told in detail).

Leader-rated disclosure intentions in study 3 were measured with three items (e.g., “The employee would openly tell me about her or his mental health problems.”) adapted from the study by Pischel and Felfe [15] and rated on a five-point Likert scale from 1 (not at all true) to 5 (completely true). Cronbach’s α was 0.95.

Sickness absence in study 1 and study 2 was measured with one item (“Please enter the number of days you have been unable to work (sickness absence) in the last two months.”).

Before conducting the main statistical analyses, we tested the measurement model because adapted or shortened scales (staff care, disclosure intentions) were used in study 1 and study 3. A confirmatory factor analysis (CFA) was conducted with MPLUS (Version 8) in study 1 to assess whether the two constructs could be empirically distinguished and whether a two-factor structure was appropriate. Guidelines generally recommend at least 5–10 cases per estimated parameter or a minimum total sample size of 100–200 participants to ensure stable estimation [49,50]. In study 3, the sample size clearly fell below the recommended threshold, and we therefore did not conduct a CFA to avoid unstable or misleading estimates.

## 4. Results

The CFA for study 1 indicated a mediocre to acceptable model fit (see Table S4). All items loaded significantly on their respective factors, with standardized loadings ranging from 0.63 to 0.96. To further evaluate the factorial structure, the two-factor model was compared with a one-factor alternative, which showed a significantly poorer fit (Δχ^2^ (7) = 655, *p* < 0.001). These results support the postulated two-factor structure and provide an empirical foundation for the subsequent analyses.

We conducted all our analyses with SPSS (Version 29.0.1.0). For the moderation analyses (Model 1) we used the PROCESS macro (Version 5.0, see https://www.processmacro.org/index.html (accessed on 13 August 2025)) for SPSS to test H1_1,2,3_, H2_1,2,3_, H3_1,2_, and H4_1,2_. For the employee perspective, it was necessary to perform the statistical analysis for H1_1,2_ and H2_1,2_ separately, as disclosure intentions and actual disclosure reflect different constructs measured with different items. Since the sickness absence measure was identical in both employee samples, we additionally tested H4 using sample membership (predominantly healthy vs. psychologically affected employees) as a dichotomous moderator to determine whether employees’ health level or deterioration better explained the moderation effect. Descriptive statistics and correlations are shown in Table 1 (study 1, study 2) and Table 2 (study 3).

First, for the employee perspective, staff care was positively related to disclosure in both studies (study 1: *B* = 0.56, *SE* = 0.08, *t* = 6.85, *p* < 0.001; study 2: *B* = 0.41, *SE* = 0.07, *t* = 6.18, *p* < 0.001), supporting H1_1,2_. As an additional descriptive analysis, we calculated conditional percentage frequencies. Conditional frequencies indicated that about 20% of employees with low staff care intended to or had disclosed their mental health issues, compared to 64–80% with high staff care (see Table S2). From the leader’s perspective, staff care was also positively related to disclosure intentions (study 3: *B* = 0.66, *SE* = 0.14, *t* = 4.81, *p* < 0.001), supporting H1_3_. Effect sizes were comparable (β = 0.61 in study 1 vs. β = 0.53 in study 3), though the leader-reported effect appeared slightly weaker. Conditional frequencies indicated that leaders with low staff care expected medium to high disclosure more often (53%) than employees reported (21%), whereas for high staff care, perceptions were closely aligned (85% vs. 79%, see Table S2).

**Figure 2:**
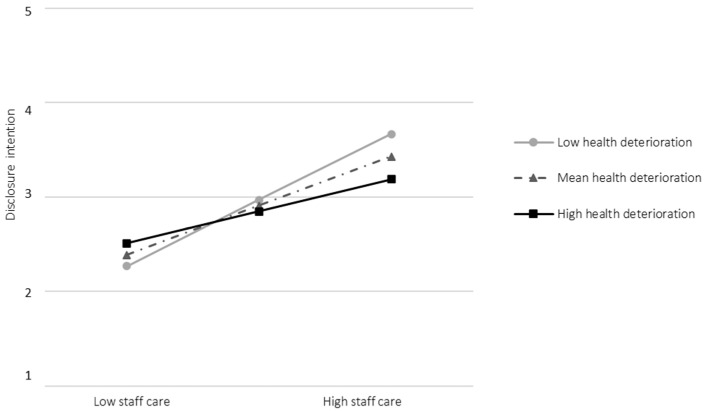
Interaction effect between staff care (Low vs. High) and health deterioration (low vs. high) on disclosure intentions to the leader (study 1). *Note.* Conditional effects are both significant (*p* < 0.01).

Second, for the employee perspective, health deterioration moderated the relationship between staff care and disclosure intentions (study 1: *B* = −0.27, *SE* = 0.12, *t* = −2.30, *p* = 0.023, see Table S1), but narrowly missed significance for actual disclosure (study 2: *B* = 0.18, *SE* = 0.09, *t* = 1.95, *p* = 0.052, see Table S1 and Figure S1). Unexpectedly, the association between staff care and disclosure intentions was stronger at low (*B* = 0.76, *SE* = 0.12, *t* = 6.26, *p* < 0.001, 95% CI [0.52, 1.00]) than at high levels of health deterioration (*B* = 0.37, *SE* = 0.12, *t* = 3.20, *p* = 0.002, 95% CI [0.14, 0.59], see Figure 2). Thus, H2_1_ was partially supported, whereas H2_2_ was not. From the leader’s perspective, the interaction narrowly missed significance (*B* = −0.25, *SE* = 0.13, *t* = −1.92, *p* = 0.058, see Table S1 and Figure S2), rejecting H2_3_. Simple slopes revealed notable descriptive differences between the employee and leader perspectives: leaders with low staff care still tended to expect a moderate likelihood of disclosure among employees with high health deterioration, whereas these employees themselves reported intentions to conceal (see Figure 2 and Figure S2).

Third, staff care was unrelated to sickness absence in the predominantly healthy sample (study 1: *B* = −0.54, *SE* = 0.38, *t* = −1.39, *p* = 0.166) but negatively related among psychologically affected employees (study 2: *B* = −1.62, *SE* = 0.56, *t* = −2.89, *p* = 0.004; see Table S1), supporting H3_2_ but not H3_1_. Conditional frequencies showed that 13% of healthy employees and 50% of affected employees with low staff care reported ≥7 sickness days, compared to 4% and 30%, respectively, under high staff care (Table S2).

**Figure 3:**
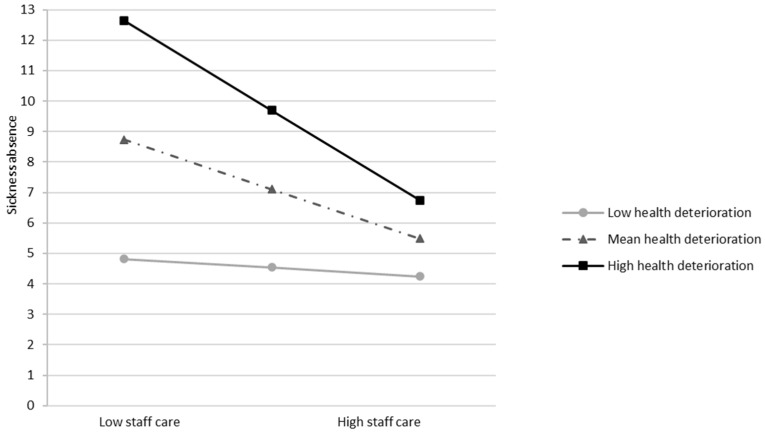
Interaction effect between staff care (Low vs. High) and health deterioration. (low vs. high) on sickness absence in days for the last two months (study 2). *Note.* Conditional effects are significant for high health deterioration (*p* < 0.001).

Fourth, health deterioration did not moderate the negative relationship between staff care and sickness absence in study 1 (*B* = −0.95, *SE* = 0.55, *t* = −1.73, *p* = 0.085; see Table S1 and Figure S3), but did in study 2 (*B* = −2.06, *SE* = 0.77, *t* = −2.66, *p* = 0.008; see Table S1). In line with our expectations, at high health deterioration, staff care showed a stronger negative association with sickness absence (*B* = −2.95, *SE* = 0.68, *t* = −4.32, *p* < 0.001, 95% CI [−4.30, −1.61]), while the effect was nonsignificant at low deterioration (*B* = −0.29, *SE* = 0.81, *t* = −0.36, *p* = 0.722, 95% CI [−1.88, 1.30]; see Figure 3). H4_1_ was rejected, whereas H4_2_ was supported.

**Figure 4:**
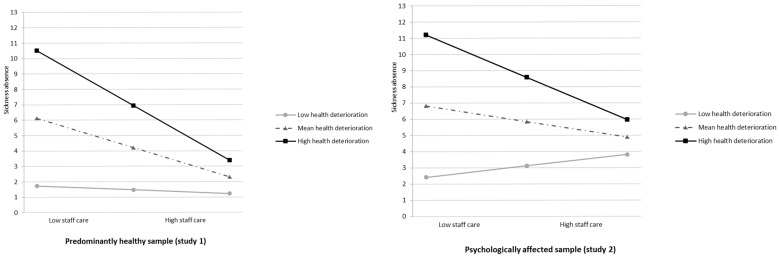
Additional analysis combining study 1 and study 2: interaction effect between staff care (low vs. high) and health deterioration (low vs. high) on sickness absence in days. *Note.* Conditional effects are significant for high health deterioration in study 1 and study 2 and mean health deterioration in study 1 (*p* < 0.001).

Additional Analysis. When combining studies 1 and 2, the interaction between staff care and sample membership was nonsignificant (*B* = 0.96, *SE* = 1.20, *t* = −0.81, *p* = 0.421; see Table S3), whereas the interaction between staff care and health deterioration remained significant (*B* = −1.92, *SE* = 0.57, *t* = −3.38, *p* < 0.001; see Table S3). Conditional effects showed that at high health deterioration, staff care was more strongly related to reduced sickness absence (study 1: *B* = −3.63, *SE* = 1.25, *t* = −2.88, *p* = 0.004, 95% CI [−6.10, −1.16]; study 2: *B* = −2.66, *SE* = 0.53, *t* = −5.07, *p* < 0.001, 95% CI [−3.69, −1.63]) while the effect was nonsignificant under low deterioration (study 1: *B* = −0.26, *SE* = 0.79, *t* = −0.33, *p* = 0.744, 95% CI [−1.81, 1.29]; study 2: *B* = 0.71, *SE* = 0.92, *t* = 0.77, *p* = 0.441, 95% CI [−1.10, 2.51]; see Figure 4). These findings are in line with study 2 and provide further support for H4.

## 5. Discussion

The high prevalence of mental health issues in the workplace poses challenges not only for affected employees but also for organizations, leaders, and teams. When employees do not feel safe to disclose their condition, and when leaders or colleagues respond with misunderstanding or rejection, problems may escalate and ultimately be accompanied by increased sickness absence. The present research examined whether health-oriented leadership (HoL), particularly staff care, is associated with employees’ disclosure (intentions) and sickness absence, especially when health deterioration occurs among previously healthy or already affected employees.

First, consistent with previous research [15], staff care was positively associated with disclosure intentions (study 1). Extending this to behavior, staff care was also associated with actual disclosure among employees with a diagnosed mental illness or severe mental health issues (study 2). Among employees perceiving high staff care, 80% reported an intention to disclose, and 64% ultimately did so. The fact that staff care was positively associated with both disclosure intentions and actual disclosure is noteworthy, as intentions do not always translate into action (the intention–behavior gap [37]). Although these associations do not imply causality, they are consistent with theoretical assumptions of the HoL and disclosure models [9,20,21]. Our findings suggest that leaders who recognize warning signals, express genuine concern for employees’ health, and initiate health-related conversations and actions may foster an atmosphere of safety and trust that reduces fear of negative consequences, thereby encouraging openness. Disclosure, in turn, may enable more timely support and appropriate workplace accommodations [14].

Second, as deteriorating mental health poses unique challenges for both leaders and employees and has been largely overlooked, we examined whether the relationship between staff care and disclosure varies depending on health deterioration. In study 1, the interaction between staff care and health deterioration was significant but, contrary to expectations, was stronger among employees with low health deterioration. This pattern can be explained in several ways. Employees imagining a disclosure situation without severe symptoms may see it more positively and perceive fewer risks of stigma, whereas those already affected may feel more vulnerable and uncertain about their leaders’ reactions, leading to hesitation even when staff care is high [24]. Leaders may also feel ambivalent when health problems become visible, as they want to respect boundaries and avoid appearing intrusive [14,17]. Moreover, factors such as the duration of symptoms or the presence of trusted confidants outside work may reduce the need to disclose to the leader and weaken the observed association at higher levels of health deterioration. Importantly, the conditional effect for high health deterioration was still significant, indicating that staff care was also linked to disclosure intentions among those already experiencing health decline (see Figure 2). In study 2, involving employees with mental illness or severe mental health issues, health deterioration did not significantly moderate the relationship between staff care and actual disclosure. However, descriptive slopes suggested that high staff care was more strongly associated with disclosure at higher deterioration levels (mean ≈ 2.9 vs. 2.4; see Figure S1). Overall, these findings indicate that, even in the presence of vulnerability and potential stigma, employees may be more willing to speak up when they perceive their leader as health-oriented.

Third, we found that staff care was negatively associated with sickness absence among employees with mental illness or severe mental health problems (study 2) but not among predominantly healthy employees (study 1). The correlation in Study 1 pointed in the expected direction (study 1). However, the correlation in study 1 pointed in the expected negative direction (see Table 1), and conditional probabilities revealed that only 4% of employees reporting high staff care had seven or more days of sickness absence, compared to 13% among those with low staff care leaders. The absence of a significant main effect in study 1 may be attributed to the limited variance in sickness absence within this sample. Approximately 86% of participants reported fewer than three sickness absence days within the two-month reference period. For predominantly healthy individuals, who tend to exhibit fewer sickness absences overall, a longer observation period may be necessary to detect more meaningful differences. This interpretation aligns with Pundt and Felfe [16], who found small negative correlations between staff care and sickness absence over a one-year period (*r* = −0.10, *p* < 0.01). Nevertheless, the percentage-based findings in our study suggest that staff care may still be practically relevant even among healthy employees, as a prolonged absence of a single worker can burden teams. Importantly, for employees already experiencing severe mental health issues, staff care seems particularly relevant, being linked to a lower likelihood and shorter duration of sickness absence. This pattern highlights the potential importance of staff care for maintaining work ability among vulnerable employees.

Fourth, we examined whether the effectiveness of staff care in relation to sickness absence varied depending on employees’ health deterioration. In study 1, the moderation was not significant, suggesting no systematic variation with health deterioration among the healthy sample. In contrast, Study 2 showed that staff care was linked to markedly lower sickness absence when employees reported high health deterioration, but not when health was stable. Combined analyses across both employee samples confirmed that this effect was driven by employees’ perceived health deterioration rather than sample membership. Specifically, when employees in either group reported worsening mental health, staff care consistently related to reduced sickness absence, whereas no effect emerged under stable health. These findings are in line with the study by Pischel & Felfe [15], who observed that the positive effect of staff care on disclosure intentions emerged independently of employees’ general mental health status. Together, the results underscore health deterioration as a critical situational factor and indicate that staff care may play a particularly meaningful role when employees experience worsening mental health, potentially mitigating further decline or extended absence.

Fifth, as prior research has shown that employees and leaders often differ in their perceptions of workplace dynamics (e.g., [32,38]), we explored whether similar discrepancies exist regarding disclosure intentions. From the leader perspective (study 3), staff care was again positively related to disclosure intentions (β ≈ 0.53), though the effect was slightly weaker than from the employee perspective in study 1 (β ≈ 0.61). The interaction between staff care and employees’ health deterioration was not significant. Nonetheless, simple slopes revealed a notable difference between perspectives: among employees with high health deterioration, leaders with low staff care still tended to expect a moderate likelihood of disclosure (see Figure S2), whereas those employees indicated an intention to conceal (see Figure 2). This mismatch may be explained by the false consensus effect [39], whereby leaders assume others share their attitudes, expecting disclosure regardless of their own health-specific behavior. Although the moderation was nonsignificant, this tendency is noteworthy because such misjudgments by leaders may reduce opportunities for timely support and inadvertently reinforce concealment or delayed help-seeking.

This study makes several theoretical contributions. First, it adds to the growing evidence of linking health-oriented leadership to positive employee outcomes [8,9] and enhances our understanding of the contextual factors that may shape its effectiveness [23]. By examining employees’ acute health deterioration as a situational contingency, the study highlights conditions under which staff care may be particularly relevant. Second, our work extends existing disclosure models (e.g., [11,20,24]) by identifying staff care as a critical factor related to disclosure.

### 5.1. Limitations and Future Directions

A first limitation concerns the cross-sectional design, which restricts causal inference and may introduce common method bias [51]. For instance, employees who are more inclined to disclose or attend work regularly may perceive their leaders as more caring, rather than this perception being shaped by staff care itself. Nevertheless, our interpretation is grounded in established theoretical frameworks, such as the HoL model [9] and existing disclosure models (e.g., [20]), and aligns with experimental findings showing causal effects of staff care on disclosure [15]. Since our research focus was on moderation effects, these are generally less affected by common method bias [52]. Furthermore, sickness absence served as an objective behavioral outcome, reducing the risk of inflation due to shared method variance. We further assume that both disclosure and sickness absence reflect employees’ current perceptions of leadership and health changes (i.e., synchronous effects [53]). Taken together, despite the limitations of the cross-sectional design, we consider our interpretations to be both theoretically grounded and empirically justified. Nevertheless, future studies should use longitudinal or experience sampling methods to provide causal evidence.

A second limitation involves the single-dyad approach in study 3, where each leader rated only one employee from their team. This approach may have introduced sampling bias or led to an overemphasis on individual experiences. It also prevents analysis of within-leader variability in expectations across different team members. For instance, if leaders selected an employee they liked or felt particularly close to, this could potentially explain why even low staff care leaders still held relatively optimistic expectations regarding disclosure. However, such bias would likely affect all leaders similarly and thus cannot fully explain our pattern of results. Despite this, including leaders’ perspectives provided valuable relational insights and revealed possible mismatches with employee views. Future research should include multiple dyads per leader to capture intra-leader variation and better account for differences in relational quality and leader perceptions across the team. Additionally, more systematic sampling procedures (e.g., random selection of employees or leader reports on the entire team) could help minimize selection bias.

A third limitation relates to the measurement validity of the reduced and adapted scales (staff care, disclosure intentions). This could have introduced measurement bias and potentially led to an underestimation or overestimation of associations. Future research should employ fully validated multi-item scales. Nonetheless, our confirmatory factor analysis in study 1 confirmed the distinctiveness of disclosure intentions and staff care. Moreover, the use of shortened scales is common in multi-construct studies, and prior research using reduced versions of staff care (e.g., [22,33]) found no evidence that descriptive statistics or correlations with related health outcomes differ meaningfully from the original scales [16]. We also used single-item measures (actual disclosure, sickness absence), which may limit the reliability and comparability. If possible, future studies may use objective organizational data (e.g., HR records) to minimize any bias. However, sickness absence represents a concrete, observable behavior rather than a latent construct requiring multiple items, and previous research has demonstrated strong agreement between self-reported and recorded sickness absence days [54]. Similarly, actual disclosure also reflects a specific behavior rather than a broader latent construct.

A fourth limitation concerns the changing nature of the work environment. Although not the central focus of this study, the increasing prevalence of remote or hybrid work introduces new dynamics for disclosure and leadership [8]. While initial findings suggest that staff care remains beneficial for employees’ health in this context, working from home also reduces informal interactions and makes it more difficult for leaders to recognize early warning signs of health deterioration and to provide timely support [8]. Consequently, the associations between staff care and disclosure identified in this study may be weaker in digital work settings, where health deterioration is less visible and communication patterns differ. Future studies should therefore explore how staff care relates to disclosure processes of employees with health declines in digital or hybrid work contexts.

A fifth limitation concerns the generalizability beyond the German context. Leadership perceptions, mental health stigma, and disclosure norms differ across cultures [34,55,56,57]. Germany’s relatively strong occupational health regulations and increasing public awareness of mental health may foster more openness and receptivity to staff care compared to countries with different workplace norms or healthcare systems. Future research should examine whether these associations hold across different cultural settings.

Despite these limitations, the inclusion of both employee and leader perspectives remains a key strength of our research, as it offers a more differentiated view of how these dynamics are reflected in workplace disclosure processes. Moreover, by studying both predominantly healthy employees (intentions) and psychologically affected employees (behavior), we were able to capture both anticipatory and real-life aspects of disclosure.

### 5.2. Practical Implications

Our findings suggest that staff care is associated with greater willingness to disclose and fewer sickness absences, particularly when employees experience health deterioration. Some leaders, however, may overestimate employees’ openness. Although the present findings do not imply causality, prior experimental research suggests that staff care can have causal effects on employee outcomes such as disclosure [15]. Given the high prevalence of mental health problems at work and evidence that many leaders feel insecure when addressing such issues [17], implementing leadership development programs that promote health-oriented behavior is crucial. These programs, grounded in the HoL model [9], should strengthen leaders’ awareness of early warning signals and their ability to respond through preventive and supportive staff care behaviors. For example, leaders can regularly check in with employees about workload and well-being, adjust tasks or schedules during demanding phases, encourage recovery breaks, or refer employees to occupational health services when needed. Established interventions, such as the comprehensive HoL intervention [58], which combine workshops, coaching, and structured feedback, may serve as practical frameworks. In addition, the Early Warning Indicator (EWI; [40]) may help leaders systematically recognize health deterioration and respond appropriately.

### 5.3. Conclusions

Taken together, the findings emphasize that staff care, as an essential component of health-oriented leadership, is associated with higher willingness to disclose mental health issues and with lower sickness absence. Staff care seems particularly relevant for supporting disclosure during early health declines and for mitigating sickness absence during acute deterioration among those already affected. These results highlight the importance of health-specific leadership behaviors, especially during phases of worsening health, when timely and supportive actions may help maintain openness and work participation.

## Figures and Tables

**Table 1:** Descriptives and correlations of variables in study 1 and study 2.

	Study 1		Study 2					
	Mean	*SD*	Mean	*SD*	1	2	3	4
1 Staff care	2.93	0.92	2.69	1.00	—	−0.11 *	0.33 ***	−0.20 ***
2 Health deterioration	1.83	0.73	3.06	0.65	−0.19 *	—	0.05	0.25 ***
3 Disclosure (intention)	2.94	1.06	2.22	1.27	0.50 ***	−0.19 *	—	0.11 *
4 Sickness absence	1.75	4.38	7.27	10.64	−0.16 †	0.20 *	−0.03	—

*Note. N*_1_ = 148, *N*_2_ = 338. Intercorrelations are presented below the diagonal for study 1, and above the diagonal for study 2. *SD* = standard deviation. † < 0.10. * *p* < 0.05. *** *p* < 0.001.

**Table 2:** Descriptives and correlations of variables in study 3.

	Mean	*SD*	1	2	3
1 Staff care	3.58	0.82	—		
2 Health deterioration	2.22	1.11	−0.03	—	
3 Disclosure intention	3.12	1.24	0.46 ***	−0.22 *	—

*Note. N*_3_ = 91. *SD* = standard deviation. * *p* < 0.05. *** *p* < 0.01.

## Data Availability

The data are available from the authors upon reasonable request. The data cannot be made publicly available due to data protection and privacy restrictions. The datasets were collected by a professional market research institute under contractual agreements that ensure participant anonymity and restrict public data sharing. Given the sensitive nature of the data, which include information on employees’ and leaders’ mental health and workplace experiences, public access could risk re-identification.

## Supplementary Materials

The following supporting information can be downloaded at: https://www.mdpi.com/article/10.3390/healthcare13212759/s1, Table S1: Regression Coefficients, Standard Errors, 95% Confidence Intervals, and Model Summary of Study 1, Study 2, and Study 3. Table S2: Conditional Percentage Frequencies Derived from Cross Table for all Studys. Table S3: Additional Analysis Combining Study 1 und Study 2: Regression Coefficients, Standard Errors, 95% Confidence Intervals, and Model Summary. Table S4: Model Fit Indices for Confirmatory Factor Analyses (CFA) in Study 1. Figure S1: Interaction Between Staff Care (Low vs. High) and Health Deterioration (Low vs. High) on Actual Disclosure to Leader (Study 2). *Note.* Conditional effects are both significant (*p* < 0.01). Figure S2: Interaction Effect Between Staff Care (Low vs. High) and Health Deterioration. (Low vs. High) on Disclosure Intentions to Leader (Study 3). Figure S3: Interaction Between Staff Care (Low vs. High) and Health Deterioration (Low vs. High) on Sickness Absence in Days for the Last Two Months (Study 1).
