# Supplementary material for: When Employee Mental Health Deteriorates: Examining the Relationship Between Health-Oriented Leadership, Disclosure, and Sickness Absence
Source: Healthcare (Basel). 2025 Oct 30;13(21):2759. doi: 10.3390/healthcare13212759 (PMC12609077; doi:10.3390/healthcare13212759)
Supplement: Supplementary file 1 [file healthcare-13-02759-s001.zip › healthcare-3854141-supplementary.pdf]

Supplementary Materials:

Table S1. Regression Coefficients, Standard Errors, 95% Confidence Intervals, and Model Summary of Study 1, Study 2, and Study 3.

Outcome	Predictors	*B*	*SE*	*t*	*p*	LLCI	ULCI
	Study 1						
Disclosure intention ($H1_1$, $H2_1$)							
	Constant	2.91	0.08	38.55	< .001	2.76	3.06
	Staff care	0.56	0.08	6.85	< .001	0.40	0.72
	Health deterioration	-0.08	0.11	-0.83	.409	-0.30	0.12
	Staff care × Health deterioration	-0.27	0.12	-2.30	.023	-0.50	-0.04
			$F(3, 144) = 19.37$, $p < .001$, $R^2 = .29$				
Sickness absence ($H3_1$, $H4_1$)							
	Constant	1.63	0.36	4.55	< .001	0.92	2.33
	Staff care	-0.54	0.38	-1.39	.166	-1.31	0.23
	Health deterioration	1.26	0.50	2.50	.014	0.26	2.26
	Staff care × Health deterioration	-0.95	0.55	-1.73	.086	-2.04	0.35
			$F(3, 144) = 3.77$, $p = .012$, $R^2 = .07$				
Study 2							
Disclosure ($H1_2$, $H2_2$)							
	Constant	2.23	0.07	34.31	< .001	2.11	2.36
	Staff care	0.41	0.07	6.18	< .001	0.28	0.54
	Health deterioration	0.16	0.10	1.54	.123	-0.04	0.36
	Staff care × Health deterioration	0.18	0.09	1.95	.052	-0.00	0.35
			$F(3, 334) = 16.01$, $p < .001$, $R^2 = .13$				
Sickness absence ($H3_2$, $H4_2$)							
	Constant	7.12	0.55	12.93	< .001	6.04	8.20
	Staff care	-1.62	0.56	-2.89	.004	-2.72	-0.52
	Health deterioration	3.99	0.86	4.66	< .001	2.30	5.67
	Staff care × Health deterioration	-2.06	0.77	-2.66	.008	-3.56	-0.55
		$F(3, 334) = 14.00$, $p < .001$, $R^2 = .11$					
Study 3							
Disclosure Intention ($H1_3$,$H2_3$)							
	Constant	3.11	0.11	27.97	< .001	2.89	3.33
	Staff care	0.66	0.14	4.81	< .001	0.39	0.94
	Health deterioration	0.33	0.11	3.13	.002	0.12	0.55
	Staff care × Health deterioration	-0.25	0.13	-1.92	.058	-0.51	0.00
			$F(3, 87) = 12.09$, $p < .001$, $R^2 = .30$				

Note. $N_1 = 148$, $N_2 = 338$, $N_3 = 91$. SE = standard error, LLCI = lower level confidence interval, ULCI = upper level confidence interval.

Table S2. Conditional Percentage Frequencies Derived from Cross Table for all Studys.

	**DV 1: Disclosure (intention)**			**DV 2: Sickness absence in the last two months**					
	Middle to high			Up to 3 days		3 to 6 days		7 or more days	
	Study 1	Study 2	Study 3	Study 1	Study 2	Study 1	Study 2	Study 1	Study 2
Low staff care (1st quartile)	20.69	23.66	53.33	77.42	35.48	9.68	15.05	12.90	49.46
High staff care (3rd quartile)	79.17	63.74	85.19	89.36	56.04	6.38	14.29	4.26	29.67

Note. Employee perspective: study 1 and study 2; Leader perspective: study 3. N1 = 148, N2 = 338, N3 = 91. Middle to high disclosure (intention) includes scale mean values of 3 or higher. Percentage frequencies for low disclosure (intentions) can be calculated by subtracting 100 with middle to high percentage frequencies.

Table S3. Additional Analysis Combining Study 1 und Study 2: Regression Coefficients, Standard Errors, 95% Confidence Intervals, and Model Summary.

**Outcome**	**Predictors**	***B***	***SE***	***t***	***p***	**LLCI**	**ULCI**
Sickness Absence (H4)							
	Constant	4.21	0.89	4.76	< .001	2.47	5.95
	Staff care	-1.94	0.92	-2.10	.036	-3.76	-0.13
	Health deterioration	3.12	0.60	5.16	< .001	1.93	4.30
	Staff care × Health deterioration	-1.92	0.57	-3.38	< .001	-3.03	-0.81
	Sample	1.64	1.13	1.45	.148	-0.58	3.87
	Staff care x Sample	0.96	1.20	0.81	.421	-1.38	3.32
		$F(5, 479) = 19.09, p < .001, R^2 = .41$					

Note. N = 485. Dichotomous moderator sample coded with 0 = Predominantly healthy sample (study 1) and 1 = Psychologically affected sample (study 2). SE = standard error, LLCI = lower level confidence interval, ULCI = upper level confidence interval.

Table S4. Model Fit Indices for Confirmatory Factor Analyses (CFA) in Study 1.

**Model**	χ^2	*df*	χ^2/df	**CFI**	**TLI**	**RMSEA**	**SRMR**	$\Delta\chi^2$	Δdf
2-Factors*	245	112	2.19	.94	.93	.090	.052		
1-Factor	900	119	7.56	.65	.60	.211	.124	655***	7

Note: N_1 = 148. CFI = Comparative Fit Index; TLI = Tucker-Lewis-Index; RMSEA = Root Mean Square Error of Approximation; SRMR = Standardized Root Mean Square Residual. *** *p* <. 001.

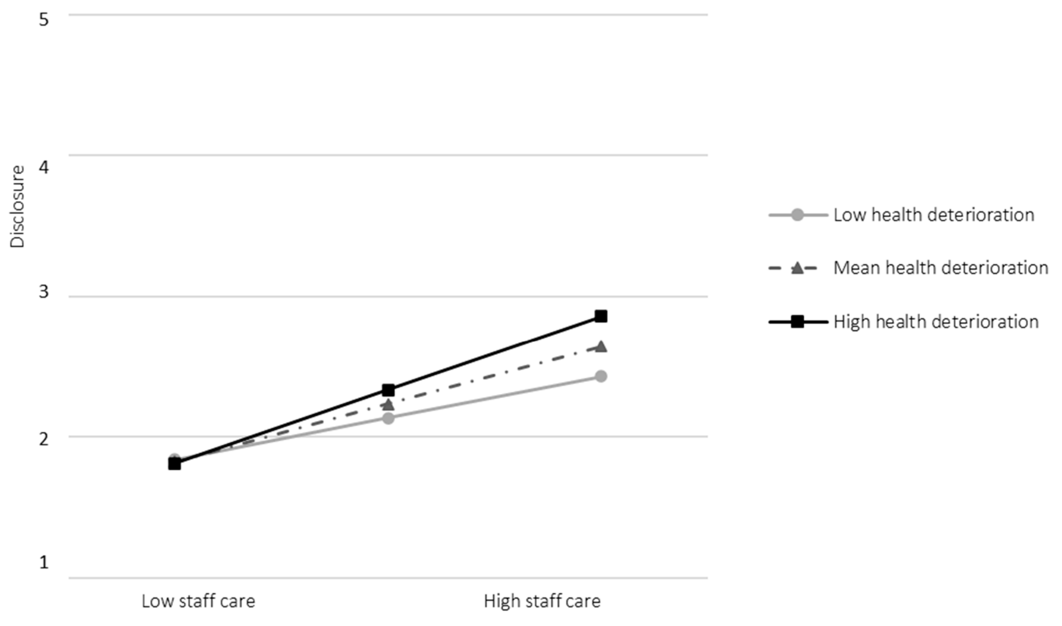

Figure S1. Interaction Between Staff Care (Low vs. High) and Health Deterioration (Low vs. High) on Actual Disclosure to Leader (Study 2). *Note.* Conditional effects are both significant ($p < .01$).

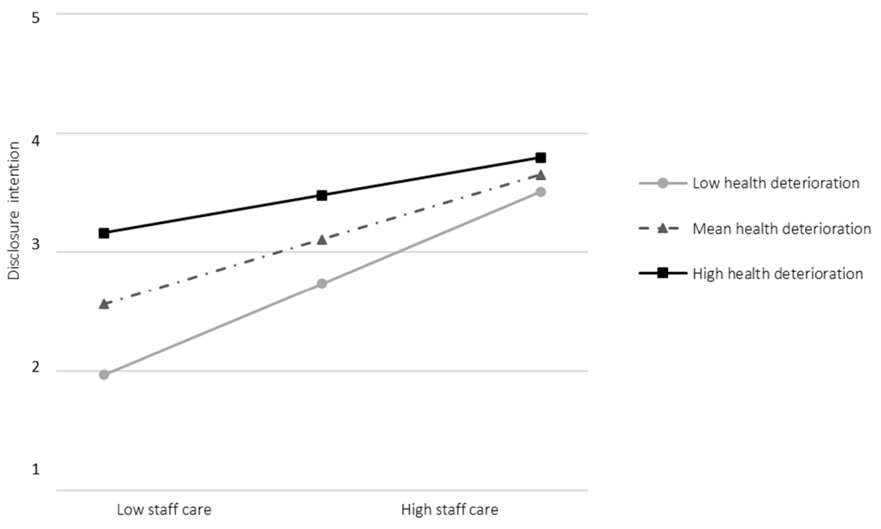

Figure S2. Interaction Effect Between Staff Care (Low vs. High) and Health Deterioration. (Low vs. High) on Disclosure Intentions to Leader (Study 3). *Note.* Conditional effects are significant for low deterioration ($p < .001$).

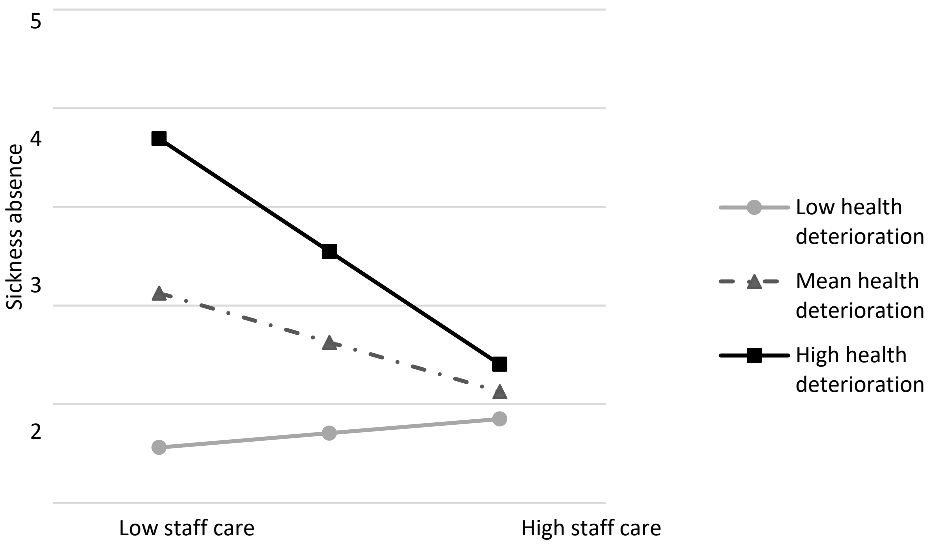

Figure S3. Interaction Between Staff Care (Low vs. High) and Health Deterioration (Low vs. High) on Sickness Absence in Days for the Last Two Months (Study 1). *Note.* Conditional effects are significant for high health deterioration (p < .001).
